# Prediction of low birth weight from fetal ultrasound and clinical characteristics: a comparative study between a low- and middle-income and a high-income country

**DOI:** 10.1136/bmjgh-2024-016088

**Published:** 2024-12-05

**Authors:** Sergio Sanchez-Martinez, Pablo Miki Marti-Castellote, Zahra Hoodbhoy, Gabriel Bernardino, Josa Prats-Valero, Ainhoa M. Aguado, Lea Testa, Gemma Piella, Francesca Crovetto, Corey Snyder, Shazia Mohsin, Ambreen Nizar, Rimsha Ahmed, Fyezah Jehan, Kathy Jenkins, Eduard Gratacós, Fatima Crispi, Devyani Chowdhury, Babar S Hasan, Bart Bijnens

**Affiliations:** 1 Department of Engineering, Universitat Pompeu Fabra, Barcelona, Spain; 2 Institut d’Investigacions Biomèdiques August Pi i Sunyer (IDIBAPS), Barcelona, Spain; 3 Department of Pediatrics and Child Health, Aga Khan University, Karachi, Pakistan; 4 BCNatal Fetal Medicine Research Center (Hospital Clínic and Hospital Sant Joan de Déu), Universitat de Barcelona, Barcelona, Spain; 5 Centre for Biomedical Research on Rare Diseases (CIBER-ER), IDIBAPS, Barcelona, Spain; 6 Cardiology Care for Children, Lancaster, Pennsylvania, USA; 7 Sindh Institute of Urology and Transplantation, Karachi, Pakistan; 8 Children's Hospital Boston, Boston, Massachusetts, USA; 9 Institut de Recerca Sant Joan de Deu, Esplugues de Llobregat, Spain; 10 ICREA, Barcelona, Spain

**Keywords:** Other diagnostic or tool, Decision Making, Obstetrics

## Abstract

**Introduction:**

Adverse perinatal outcomes (APO) pose a significant global challenge, particularly in low- and middle-income countries (LMICs). This study aims to analyse two cohorts of high-risk pregnant women for APO to comprehend risk factors and improve prediction accuracy.

**Methods:**

We considered an LMIC and a high-income country (HIC) population to derive XGBoost classifiers to predict low birth weight (LBW) from a comprehensive set of maternal and fetal characteristics including socio-demographic, past and current pregnancy information, fetal biometry and fetoplacental Doppler measurements. Data were sourced from the FeDoC (Fetal Doppler Collaborative) study (Pakistan, LMIC) and theIMPACT (Improving Mothers for a Better PrenAtal Care Trial) study (Spain, HIC), and included 520 and 746 pregnancies assessed from 28 weeks gestation, respectively. The models were trained on varying subsets of the mentioned characteristics to evaluate their contribution in predicting LBW cases. For external validation, and to highlight potential differential risk factors for LBW, we investigated the generalisation of these models across cohorts. Models’ performance was evaluated through the area under the curve (AUC), and their interpretability was assessed using SHapley Additive exPlanations.

**Results:**

In FeDoC, Doppler variables demonstrated the highest value at predicting LBW compared with biometry and maternal clinical data (AUC_Doppler_, 0.67; AUC_Clinical_, 0.65; AUC_Biometry_, 0.63), and its combination with maternal clinical data yielded the best prediction (AUC_Clinical+Doppler_, 0.71). In IMPACT, fetal biometry emerged as the most predictive set (AUC_Biometry_, 0.75; AUC_Doppler_, 0.70; AUC_Clinical_, 0.69) and its combination with Doppler and maternal clinical data achieved the highest accuracy (AUC_Clinical+Biometry+Doppler_, 0.81). External validation consistently indicated that biometry combined with Doppler data yielded the best prediction.

**Conclusions:**

Our findings provide new insights into the predictive role of different clinical and ultrasound descriptors in two populations at high risk for APO, highlighting that different approaches are required for different populations. However, Doppler data improves prediction capabilities in both settings, underscoring the value of standardising ultrasound data acquisition, as practiced in HIC, to enhance LBW prediction in LMIC. This alignment contributes to bridging the health equity gap.

**WHAT IS ALREADY KNOWN ON THIS TOPIC**

- A search on PubMed up to mid-2024 revealed that only seven studies have explored the use of machine learning to predict low birth weight (LBW), preterm birth or small for gestational age using ultrasound data, and none have focused on cohorts from low-and middle-income countries (LMICs). Existing research has largely overlooked the generalisability of these models across both high-income countries and LMICs. Additionally, no studies have assessed the added value of incorporating ultrasound data (including Doppler) alongside maternal clinical characteristics to predict LBW in resource-constrained settings.

**WHAT THIS STUDY ADDS**

- This study is among the first to develop and externally validate machine learning models for predicting LBW cases using both ultrasound data and maternal clinical characteristics in an LMIC. The validation piece evidences that maternal clinical data can be augmented with ultrasound information to predict LBW in an LMIC.

**HOW THIS STUDY MIGHT AFFECT RESEARCH, PRACTICE OR POLICY**

- LBW accounts for a significant proportion of neonatal mortality worldwide. Our findings highlight the feasibility of enhancing ultrasound practices in LMICs to improve LBW prediction and potentially narrow the health equity gap. They also underscore the need for careful application of predictive models across different settings.

## Introduction

 Despite a 53% global reduction in mortality among children under 5 years of age, slow progress has been made in decreasing stillbirths and newborn deaths.^1^ These outcomes constitute a large proportion of deaths in children under 5 years of age and include many neonates born with a low birth weight (LBW, less than 2.5 kg), which the WHO recognises as a critical global health issue.^2^ LBW can include infants who are small for their gestational age (SGA, <10th centile of weight for gestational age and sex), or those born prematurely (<37 weeks of gestation).^3^ In settings where accurate gestational age (GA) assessment is not possible, only LBW can be accurately identified, as both prematurity and SGA are GA-dependent. As a result, many research initiatives in low-and middle-income countries (LMICs) have predominantly concentrated on LBW.^4^ Apart from having a higher risk of perinatal mortality, LBW infants are at a higher risk of perinatal morbidity, poor neurodevelopment and increased cardiovascular problems as they grow.^5 6^ LBW fetuses can stem from diverse reasons for placental insufficiency, including placental abnormality, maternal cardiovascular problems, pre-eclampsia and maternal malnutrition leading to iron deficiency anaemia or protein-calorie deficiency, among others.^6^

Timely recognition combined with a comprehensive care pathway could prevent 75% of deaths among children under 5 years of age.^7^ This care should span the prenatal, intrapartum and postnatal periods for both mother and child. While this challenge exists globally, it is intensified in LMICs like Pakistan, where a fragile healthcare system contributes to delayed recognition and suboptimal management. In consequence, Pakistan has been rated by UNICEF as the ‘riskiest place’ for the birth of a child.^8^,^10^

Several maternal characteristics have been typically used to identify at-risk pregnancies,^11^ such as socio-demographic information, past obstetrical history and current pregnancy health indicators.^12^ In addition, the fetal heart is known to adapt to adverse intrauterine environments, such as uteroplacental insufficiency, leading to compensatory mechanisms like cardiac remodelling and blood flow redistribution across fetal vessels.^13^ Consequently, Doppler ultrasound on the fetoplacental circulation has recently emerged as a crucial technique to predict fetal growth restriction and neonatal morbidity and mortality.^14^ Lastly, B-mode ultrasound imaging is used to measure fetal biometry (abdominal circumference (AC), head circumference (HC), biparietal diameter (BPD) and femur length (FL)), which provides valuable information to detect fetal (abnormal) growth.^15^ Even with the known predictive power of these variables, our capacity to identify ‘at risk’ LBW fetuses, when no signs or symptoms have yet manifested, is limited, emphasising the need for technologically advanced solutions.^16^

Machine learning (ML) approaches could offer significant advancements in this field and have already demonstrated their relevance in predicting fetal growth restriction using fetal ultrasound^17^ and routine cardiotocography.^18^ XGBoost models, in particular, have shown effectiveness in predicting LBW cases based solely on maternal clinical characteristics.^19^ Their capacity to integrate and interpret large amounts of complex heterogeneous data hold promise to predict at-risk cases where timely interventions can improve outcomes.^20 21^ Particularly in LMIC, this approach could help close the health equity gap.

The primary objective of this study was to develop, and externally validate, ML models that can leverage maternal clinical characteristics, fetal Doppler flow measurements and fetal biometry to identify LBW fetuses from distinct high-risk cohorts, one from an LMIC and another from a high-income country (HIC). The same ML model development approach was then followed to predict occurrences of SGA cases, preterm births, caesarean sections and perinatal deaths. Providing caregivers in resource-constrained settings with such advanced technologies would enable pregnancy risk stratification, thereby allowing timely interventions for cases at higher risk.^22^

## Methods

### Study setting and participants

The first of the two studies included the FeDoC (Fetal Doppler Collaborative) study, a prospective community-based observational cohort study that took place in a peri-urban settlement of Karachi, Pakistan, from 2018 to 2019 (ClinicalTrials.gov Identifier: NCT03398551). Even though community-based, this cohort is considered at a higher risk for LBW compared with an HIC, given the expected high prevalence of LBW cases in Pakistan.^2 23^ The rationale and detailed study procedures have been described earlier.^24^ Participants included 694 pregnant women between 22 and 34 weeks of gestation who signed a written informed consent.

The second cohort was sourced from the IMPACT (Improving Mothers for a Better PrenAtal Care Trial) study (ClinicalTrials.gov Identifier: NCT03166332), which is a randomised controlled clinical trial that took place at the Hospital Clínic of Barcelona, Spain, from 2017 to 2020. The 1221 participants were pregnant women, over 18 years old. The definition of high risk for this study follows the Royal College of Obstetricians and Gynaecologists (RCOG) guidelines.^25 26^ These women were randomly allocated to three arms of intervention: a Mediterranean diet, a mindfulness-based stress reduction programme or no intervention. Details are described in a study by Crovetto *et al*.^26^ All individuals who agreed to participate provided written informed consent before being enrolled.

Both cohorts underwent standardised ultrasound studies during the study period. No women participated twice in these studies, even if pregnant more than once during the recruitment period.

### Data selection and processing

This study included pregnancies with third-trimester fetoplacental ultrasound data available. The analysis focused on data variables present in both cohorts, and all clinical and imaging measurements were collected after 28 weeks of gestation. The variables comprised a total of five distinct categories: maternal socio-demographic information, past obstetrical history, current pregnancy health indicators, fetoplacental Doppler and fetal biometry. Pulsatility indices (PI) for the umbilical artery (UA) and middle cerebral artery (MCA) were extracted from focused Doppler pulsed-wave acquisitions. These were calculated as the difference between the peak systolic flow and minimum diastolic flow velocity, divided by the mean velocity recorded throughout the cardiac cycle. Cerebro-placental ratio (CPR) was calculated by dividing MCA PI by UA PI. Additionally, fetal biometry was obtained from routine ultrasound examination including BPD, HC, AC and FL, each of which was acquired thrice and averaged. In IMPACT, GA was estimated based on the crown-rump length (CRL) measured during the first trimester. In FeDoC, no first-trimester ultrasound was available, and GA was determined using the Hadlock formula that considers BPD, HC, AC and FL.^27^

Observations missing continuous variables were removed from the analyses and those containing missing entries for binary variables were set to 0 (assuming that the event did not occur and was not recorded). The proportion of missing data is provided in supplemental material (online supplemental tables S1A, B). To harmonise the data sets, we standardised key variables by creating corresponding categories across both FeDoC and IMPACT. This involved mapping variables such as high-risk behaviours (smoking, tobacco use, betel nut chewing, alcohol consumption, drug use), education level and employment status to these newly established categories, ensuring consistency between the data sets despite differences in the original data formats. To account for the influence of GA in biometry and Doppler information, the analyses incorporated the GA at the ultrasound visit. Details on the data harmonisation are included in the online supplemental material.

### Outcomes and reference standards

The FeDoC and IMPACT studies primarily investigated stillbirth or early neonatal mortality and SGA, respectively. These studies had secondary outcomes encompassing a wide array of adverse perinatal outcomes (APO), which included conditions such as severe fetal growth restriction, preterm birth (defined as delivery prior to 37 weeks gestation^28^) and LBW.

In this analysis, the main focus was on predicting pregnancies classified as LBW, defined as birth weight less than 2.5 kg.^2^ As a secondary objective, models were developed to predict SGA, which was defined as birth weight below the 10th centile according to The International Fetal and Newborn Growth Consortium for the 21st Century (INTERGROWTH-21st) standard^29^ in both cohorts. Additionally, for the IMPACT study, SGA was identified using a local Spanish standard developed in 2007,^30^ which is tailored to regional characteristics. The differences in predictive performance arising when comparing the two standards are discussed in the supplementary material (online supplemental table S2, figure S1). Lastly, the value of the interrogated data for predicting preterm births, perinatal deaths (defined as stillbirth or neonatal mortality within 7 days of life, only present in FeDoC) and caesarean section occurrences were also assessed.

### Statistical analysis

Differences between the characteristics of the two studies were evaluated using a t-test or Mann-Whitney U test for continuous variables, depending on the normality of the data, as assessed by the Kolmogorov-Smirnov test. The χ^2^ test was used for binary variables. After Bonferroni correction, a p value<0.001 was considered significant for each of these comparisons.

### Predictive models

**Figure 1:**
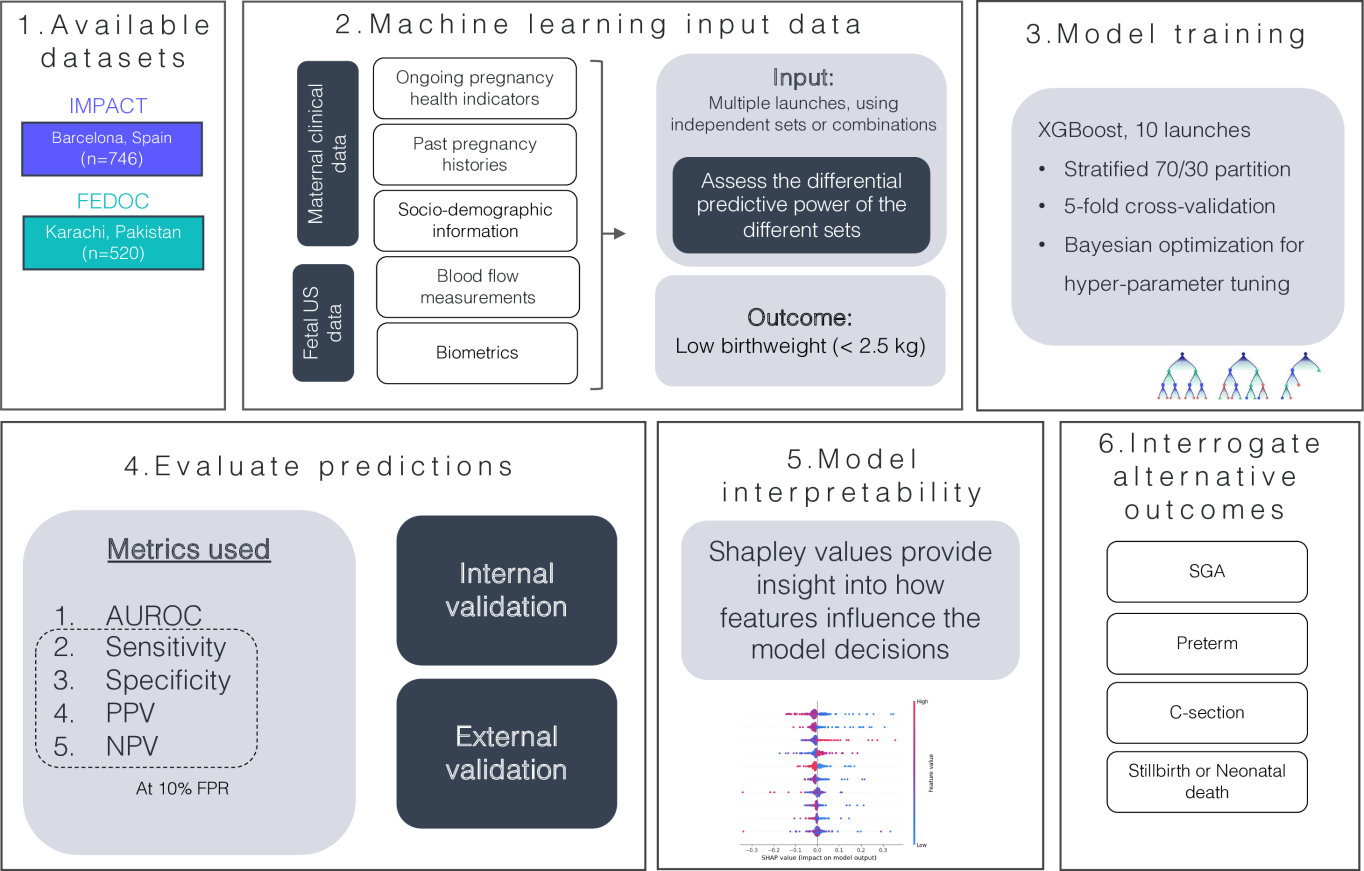
Diagram of the methodology followed in this work. AUROC, area under the receiver operating characteristic; FeDoC, Fetal Doppler Collaborative; FPR, false positive rate; IMPACT, Improving Mothers for a Better PrenAtal Care Trial; NPV, negative predictive value; PPV, positive predictive value; SGA, small for gestational age; SHAP, SHapley Additive exPlanations; US, ultrasound.

A diagram illustrating the methodology followed in this work is presented in figure 1. To identify the most predictive factors for LBW, several supervised gradient-boosted ensemble models were developed using the XGBoost Python implementation.^31^ The models were initialised using a class-weighting strategy to account for class imbalance and used the receiver operating characteristic area under the curve (ROC AUC) as a measure of performance. The two cohorts were divided into 70% training and 30% test sets, with stratification based on the outcome being analysed. Model development was done following fivefold cross-validation, coupled with Bayesian optimisation for hyperparameter tuning, which is standard practice given the sample size of the analysed cohorts. No feature selection strategy was implemented, but Lasso and Ridge regularisation were implemented during the model’s training, which effectively penalise the model for using irrelevant or redundant features (decreasing bias and variance), thus reducing overfitting, and improving the model’s generalisation.

Given the limited number of studies and the class-imbalance scenario, the model’s stability and performance were evaluated over 10 iterations using different randomised train/test splits. The metrics used for evaluation were average sensitivity, specificity, positive predictive value, negative predictive value (calculated at a 10% false-positive rate, as commonly reported in clinical literature for SGA prediction^32^) and AUC, along with their corresponding SD. For further interpretation of results, only the best models (out of 10 iterations) were subsequently assessed using SHapley Additive exPlanations (SHAP).^33^ These models are assumed to be the ones that best capture the underlying data patterns and associations with the interrogated outcome. SHAP values elucidate these associations by illustrating the contribution and influence of each variable on the model’s prediction.

Considering the different risk factors associated with LBW, the training of the models was explored with differing sets of relevant features and their combinations, to analyse the differential power they brought to the prediction. The data included in each set is detailed in table 1. For validation purposes, the defined sets were identical for both cohorts.

**Table 1:** Overview of data included in each set for both cohorts

	Maternal clinical data	Fetal biometry	Doppler
Set 1	✓		
Set 2		✓	
Set 3			✓
Set 4	✓	✓	
Set 5	✓		✓
Set 6		✓	✓
Set 7	✓	✓	✓

This table should be read horizontally. A tick mark indicates that a certain data source (column) is included in the specified data set (row).

We performed experiments to evaluate the models’ performance through both internal and external validation. Internal validation involved training and testing the models within the same cohort, while external validation involved training on one cohort and testing on the other to assess the models’ generalisability across different cohorts. This dual approach enabled us to explore potential differential risk factors for the outcomes examined.

Finally, the exact same procedure was repeated for the secondary outcomes, including SGA, preterm birth, caesarean section and perinatal death.

We have adhered to the Transparent Reporting of a multivariable prediction model for Individual Prognosis or Diagnosis (TRIPOD) statement.^34^ The TRIPOD checklist is provided in the online supplemental file 1.

## Results

Among the 694 participants in the FeDoC study and the 1221 participants in the IMPACT trial, our study included only 520 and 746 individuals, respectively. These participants comprised women assessed from 28 weeks of gestation, with complete maternal and fetal characteristics. Online supplemental figure S2 shows the flow of participants in each study. Table 2 presents the maternal and fetal characteristics of the study populations, including all predictive variables and outcome data interrogated by the ML models for prediction purposes.

**Table 2:** Maternal and fetal characteristics of the study populations

Variables	FeDoC (n=520)	IMPACT (n=746)	P value
**Maternal socio-demographics**			
Maternal age (years)	28.0 (23.0–30.0)	37.4 (34.5–40.5)	<0.001
Work status			
Unemployed	507 (97.5)	50 (6.7)	<0.001
Self-employed	7 (1.4)	55 (7.4)	<0.001
Private/student/other employment	6 (1.1)	641 (85.9)	<0.001
Education level			
No or primary education	397 (76.3)	27 (3.6)	<0.001
Secondary or technology education	117 (22.5)	219 (29.4)	0.007
University education	6 (1.1)	500 (67.0)	<0.001
Unhealthy habits			
No unhealthy habits	257 (49.4)	437 (58.6)	0.003
Stopped after pregnancy confirmation	9 (1.7)	256 (34.3)	<0.001
Continued during pregnancy	254 (48.8)	53 (7.1)	<0.001
**Current pregnancy health indicators**			
Maternal height (cm)	154.8 (±5.7)	163.5 (±6.3)	<0.001
Maternal weight (kg)	57.5 (51.2–65.8)	72.8 (65.7–81.7)	<0.001
Maternal body mass index (kg/m^2^)	24.0 (21.8–27.3)	27.1 (24.8–30.3)	<0.001
Maternal SBP at visit time (mm Hg)	107.0 (99.0–114.2)	108.0 (100.2–115.0)	0.016
Maternal DBP at visit time (mm Hg)	70.0 (64.0–75.0)	71.0 (66.0–77.0)	<0.001
Maternal Hb at visit time (g/dL)	9.08 (8.1–10.1)	11.7 (11.1–12.3)	<0.001
Antenatal care access	372 (71.5)	746 (100.0)	<0.001
**Complications** (self-reported in FeDoC)			
Hypertensive pregnancy disorders*	29 (5.6)	83 (11.1)	<0.001
Convulsions	1 (0.19)	1 (0.13)	1†
Gestational diabetes mellitus	11 (2.1)	67 (9.0)	<0.001
Anaemia or iron deficiency	234 (45.0)	295 (39.5)	0.060
Fever or antibiotic use	167 (32.1)	64 (8.6)	<0.001
Pregnancy-related bleeding	25 (4.8)	34 (4.6)	0.942
**Past pregnancy histories** (self-reported in FeDoC)			
History of normal previous pregnancies	424 (81.5)	464 (62.2)	<0.001
History of previous preterm births	156 (30.0)	36 (4.8)	<0.001
History of previous fetal deaths (including abortions)	108 (20.8)	304 (40.7)	<0.001
**Fetal growth**			
GA at ultrasound (weeks)	31.2 (30.4–32.6)	33.3 (32.4–34.1)	<0.001
GA at clinical data collection (weeks)	31.2 (30.4–32.6)	35.0 (34.0–36.0)	<0.001
Head circumference (cm)	28.6 (27.9–29.8)	30.3 (29.3–31.2)	<0.001
Abdominal circumference (cm)	27.5 (26.5–29.0)	29.2 (28.0–30.5)	<0.001
Biparietal diameter (cm)	8.0 (7.8–8.3)	8.3 (8.0–8.6)	<0.001
Femur length (cm)	6.1 (5.9–6.4)	6.3 (6.1–6.5)	<0.001
**Fetoplacental blood flow measurements**			
Middle cerebral artery pulsatility index	1.85 (1.58–2.15)	1.89 (1.68–2.13)	0.091
Umbilical artery pulsatility index	1.11 (0.98–1.25)	0.94 (0.84–1.06)	<0.001
Cerebro-placental ratio	1.67 (1.38–2.01)	2.00 (1.71–2.35)	<0.001
**Outcomes**			
Low birth weight (<2.5 kg)	94 (18.1)	48 (6.4)	<0.001
SGA (BW<10th centile as per IG21)	87 (16.7)	78 (10.5)	0.001
Preterm (<37 weeks)	141 (27.1)	24 (3.2)	<0.001
Caesarean section	93 (17.9)	242 (32.4)	<0.001
Perinatal death	33 (6.3)	0 (0.0)	<0.001

Continuous variables with a normal distribution are reported as mean±SD, with differences assessed using the t-test. For non-normally distributed continuous variables, values are presented as the median and IQR, with differences assessed using the Mann-Whitney U test. Binary variables are reported as counts and percentages, and their differences are evaluated using the χ2 test. Unhealthy habits encompass smoking, sniffing/chewing tobacco, chewing betel nut, alcohol consumption and drug use.

* Hypertensive pregnancy disorder is defined as systolic blood pressure ≥140 mm Hg or diastolic blood pressure ≥90 mm Hg measured at least 4 hours apart.

† Yates correction produced a p value of 1.

BW, birth weight; DBP, diastolic blood pressure; FeDoC, Fetal Doppler Collaborative; GA, gestational age; Hb, haemoglobin; IG21, INTERGROWTH-21st; IMPACT, Improving Mothers for a Better PrenAtal Care Trial; INTERGROWTH-21st, The International Fetal and Newborn Growth Consortium for the 21st Century; SBP, systolic blood pressure; SGA, small for gestational age.

The socio-demographic differences mirror the distinctions between LMIC and HIC. The unemployment rate in FeDoC was close to 97.5%, compared with only 6.7% in IMPACT. Educational disparities were also stark: 76.3% of women in FeDoC did not attend school or only completed primary education, whereas 96.4% of women in IMPACT completed at least secondary education. Pregnant women in FeDoC were younger, shorter, underweight and had lower haemoglobin levels. Additionally, up to 30% of them did not have access to antenatal care. There were statistically significant differences in past pregnancy variables and complications during the current pregnancy. GA at scan was significantly different, and so were fetal growth characteristics and fetoplacental blood flow measurements. Lastly, there were significant differences in all outcomes studied.

In table 3 we show the mean and SD, resulting from 10 launches, when training with each feature set and with each combination of internal (within-data set) and external validation scenarios. During internal validation within FeDoC, Doppler data stood out as the single most predictive variable set, over maternal clinical data and biometry (AUC_Doppler_, 0.67; AUC_Clinical_, 0.65; AUC_Biometry_, 0.63), with the combination of clinical and Doppler information proving to be the most effective for prediction (AUC_Clinical+Doppler_ of 0.71). During internal validation within IMPACT, biometry stood out as the single most predictive variable set (AUC_Biometry_, 0.75; AUC_Doppler_, 0.70; AUC_Clinical_, 0.69), with its combination with Doppler and maternal clinical data achieving the highest prediction accuracy (AUC_Clinical+Biometry+Doppler_ of 0.81). External validation scenarios indicated a consistent trend of Doppler as the single most predictive set (from FeDoC to IMPACT: AUC_Doppler_ 0.73; AUC_Biometry_, 0.63; AUC_Clinical_, 0.59. From IMPACT to FeDoC: AUC_Doppler_ 0.70; AUC_Biometry_, 0.62; AUC_Clinical_, 0.57) and paired with biometry data remained as the most predictive combination (from FeDoC to IMPACT: AUC_Biometry+Doppler_, 0.75. From IMPACT to FeDoC: AUC_Biometry+Doppler_, 0.71), implying that maternal clinical data might not significantly contribute to the transferability of the models across cohorts.

**Table 3:** Low birth weight prediction area under the curves for the different sets of features

		Set 1	Set 2	Set 3	Set 4	Set 5	Set 6	Set 7
		Clinical	Biometry	Doppler	Clinical Biometry	Clinical Doppler	Biometry Doppler	Clinical Biometry Doppler
**Train:**	FeDoC	0.65±0.05	0.63±0.04	0.67±0.04	0.67±0.04	**0.71±0.04**	0.67±0.06	0.70±0.05
**Test:**	FeDoC							
**Train:**	IMPACT	0.69±0.09	0.75±0.04	0.70±0.07	0.77±0.05	0.71±0.06	0.78±0.03	**0.81±0.04**
**Test:**	IMPACT							
**Train:**	FeDoC	0.59±0.06	0.63±0.11	0.73±0.07	0.58±0.05	0.67±0.07	**0.75±0.06**	0.72±0.08
**Test:**	IMPACT							
**Train:**	IMPACT	0.57±0.07	0.62±0.05	0.70±0.05	0.61±0.05	0.68±0.08	**0.71±0.05**	0.69±0.05
**Test:**	FeDoC							

In each of the internal and external validation scenarios, the results of the set featuring the highest predictive performance are highlighted in bold.

FeDoC, Fetal Doppler Collaborative; IMPACT, Improving Mothers for a Better PrenAtal Care Trial.

**Figure 2:**
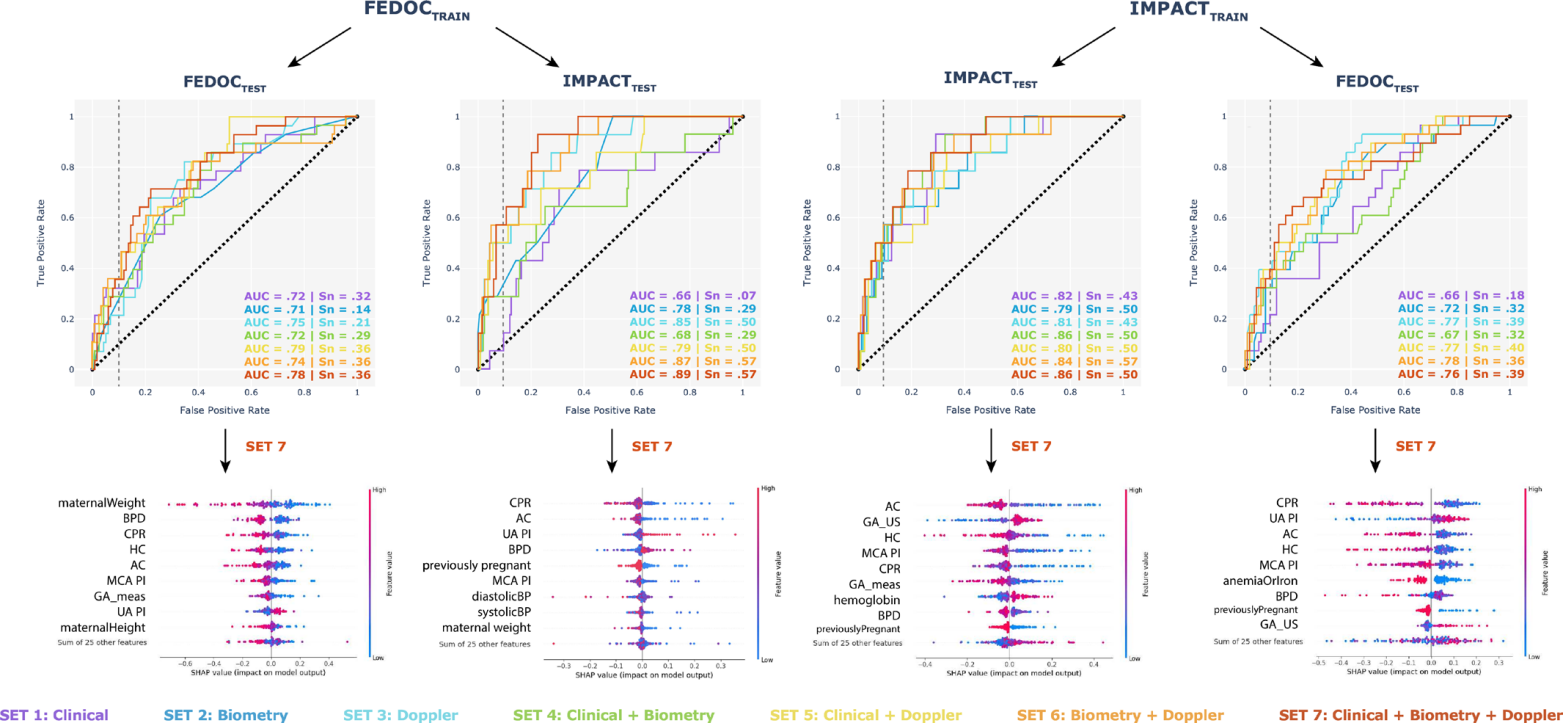
Accuracy metrics and feature interpretation of the models that achieved the best performance across iterations. The first row displays ROC curves for predictions done within (internal validation) and across (external validation) data sets for each feature set. Dotted vertical lines on each ROC plot represent a 10% false positive rate, and their intersection with each ROC curve indicates the true positive rates (sensitivity scores) reported in the figure. Below these, SHAP values corresponding to the model using the full feature set (Set 7) are reported. Variables are ranked top to bottom according to their impact on model output. Red or blue dots represent higher or lower values of inputs, respectively. AC, abdominal circumference; AUC, area under the curve; BP, blood pressure; BPD, biparietal diameter; CPR, cerebro-placental ratio; FeDoC, Fetal Doppler Collaborative; FL, femur length; GA_meas, gestational age at clinical data collection; GA_US, gestational age at ultrasound scan; HC, head circumference; IMPACT, Improving Mothers for a Better PrenAtal Care Trial; MCA PI, Middle Cerebral Artery Pulsatility Index; ROC, receiver operating characteristic; SHAP, SHapley Additive exPlanations; Sn, sensitivity; UA PI, Umbilical Artery Pulsatility Index.

We present ROC curves and SHAP values for the models that achieved the best performance across launches in figure 2. SHAP values identified the features exerting the most influence on model decisions, which only exhibited subtle variations in within-data set and external validation scenarios. When training on FeDoC, maternal weight and height appear at the top of the ranking, together with ultrasound data, which highlights the importance of maternal clinical characteristics in this population, as reported in table 3. The associations between SHAP values and LBW decisions remained consistent across scenarios and confirmed existing clinical knowledge. A positive SHAP value indicates that the feature (whose value is indicated by the colormap in figure 2) increases the likelihood of LBW, while a negative SHAP value indicates a decreased likelihood. Specifically, lower fetal biometry measurements, MCA PI, CPR, lower maternal height and weight and no history of previous pregnancies, were indicative of an elevated risk of LBW. Conversely, a higher GA at the time of ultrasound and higher UA PI were associated with a greater likelihood of LBW occurrence.

In the supplementary material, we provide a comprehensive report on the training versus test classification accuracies, along with other classification metrics obtained at a 10% false positive rate, including sensitivity, specificity, positive predictive value and negative predictive value (online supplemental table S3). Additionally, the supplement contains a detailed report of the results obtained for all secondary outcomes assessed (online supplemental table S4-9). In summary, the best prediction for SGA was obtained when combining all clinical and ultrasound data during internal validation (within FeDoC: AUC 0.73; within IMPACT: AUC 0.80). During external validation, the best result when training in FeDoC and testing on IMPACT was achieved with biometry plus Doppler data (AUC 0.77), while from IMPACT to FeDoC, the best prediction came from combining all data sources (AUC 0.71). The best prediction for caesarean sections during internal validation used maternal clinical characteristics alone (within FeDoC: AUC 0.63; within IMPACT: AUC 0.65). During external validation, all predictions were below AUC 0.60. Predictions for spontaneous preterm deliveries (excluding caesarean sections) generally had AUCs below 0.60. For perinatal death (only available in FeDoC), predictions were around AUC 0.50.

## Discussion

To our knowledge, this study pioneers the use and comparison of ML models trained on a variety of Doppler and B-mode ultrasound fetal data, along with maternal clinical characteristics, to predict LBW fetuses in an LMIC. Moreover, the development of the same models in a high-risk high-income setting allows investigating potential setting-specific causes for increased risk as well as evaluating performance in external validation across these two settings.

The healthcare disparities, such as variations in access to prenatal care witnessed among the two cohorts, underline the influence of demographic, socioeconomic and healthcare factors on the outcomes of pregnancies and neonatal health. Understanding these differential baseline characteristics is crucial when interpreting and applying (ML-based) predictive models across geographically and income-diverse populations (see online supplemental figure S3).

During internal validation within IMPACT, the most informative combination in predicting LBW cases included biometry and haemodynamic Doppler descriptors, which confirms what is currently suggested in clinical guidelines.^15^ The prediction accuracy obtained with this combination (AUC, 0.81) is comparable to current clinical practice.^32 35^ During internal validation within FeDoC, Doppler data stood out as the single most predictive variable set, whose predictive capability was augmented with the addition of maternal information (AUC, 0.71). This underscores the value of ultrasound data to predict LBW cases in this setting beyond maternal clinical characteristics. The external validation scenarios highlighted that the substantial difference in maternal clinical data between cohorts rendered these data inconsequential for the transferability of the models. Noteworthy, the combination of biometry and Doppler information yielded the highest prediction accuracy in external validation, matching the predictions observed during internal validation within the same cohort (table 3, Set 6 results).

Regarding the secondary outcomes assessed, the prediction of SGA showed moderate to good performance both within-data set and in external validation cases and was comparable to the LBW results reported in table 3. The prediction of preterm deliveries had a moderate performance within the IMPACT data set but dropped to random chance when excluding cases due to caesarean section.

The different classification performances for LBW obtained during internal validation within FeDoC (AUC, 0.71) and IMPACT (AUC, 0.81), may be due to different risk factors or disease processes for LBW among the analysed cohorts. In IMPACT, the higher predictive value of biometry and Doppler data, likely reflects the haemodynamic alterations leading to placental insufficiency (true growth-restricted fetuses). However, in FeDoC, the higher weight given by models to maternal characteristics points to risk factors linked to maternal causes, with somehow less haemodynamic changes.

Another interesting aspect is the value of fetal biometry for the prediction of LBW. While it would seem straightforward that fetal biometry at scan should contribute to predicting fetal weight at birth, as it is confirmed in IMPACT, in FeDoC it seems not to provide information beyond Doppler and maternal clinical data. We hypothesise that LBW might be more related to high (maternal) clinical risk and less related to haemodynamic changes. In this low-resource setting, traditional biometric markers combining bone sizes and abdominal circumference might not fully capture the effect of, for example, maternal malnutrition, maternal cardiac maladaptation at the third trimester or other pregnancy problems.

When comparing our work to the existing literature, results can vary significantly depending on study population characteristics, cohort size, the parameters included and the complexity of the ML models used. For instance, studies from LMICs like Ethiopia,^36^ India^37^ and Bangladesh,^38^ employed different models, predictors and sample sizes, making direct comparisons challenging. Some reported lower performance, while others demonstrated predictive abilities comparable to those in our study. However, none of these studies incorporated ultrasound data for LBW prediction. Similarly, studies from higher-income countries, such as Iran^19^ and the United Arab Emirates,^39^ showed comparable predictive performances but also did not use ultrasound information. In contrast, previous research demonstrating the predictive power of ultrasound-derived data, such as biometry and Doppler, has primarily focused on higher-income populations.^40^,^42^ The novelty of our work lies in using both clinical and ultrasound data to contrast HIC and LMIC settings, and in performing external validation across these diverse cohorts.

### Strengths, limitations and research opportunities

To the best of our knowledge, this study is among the first to develop a ML model for the prediction of LBW cases using ultrasound data and maternal clinical characteristics in an LMIC. The developed models behave similarly in the analysed settings, suggesting that there is value in ultrasound data to predict LBW in an LMIC beyond the maternal clinical characteristics typically used in these resource-constrained settings. Our findings drawn from the FeDoC study demonstrate the feasibility of aligning ultrasound data acquisition and quality with practices in HIC, which would potentially narrow the health equity gap.

Among the limitations of our study, the interrogation of such distinct cohorts to externally validate our ML models and identify potential differential risk factors for LBW, the limited sample size and the fact that these were all high-risk women render our work a valid proof of concept. However, our findings should be considered preliminary and may lack generalizability. Additionally, while both cohorts exhibited an elevated risk for APOs, the FeDoC study was community-based, whereas the IMPACT study was a multiarm clinical trial with stringent selection criteria. This challenge is common in global health research, where it is often necessary to contrast data from diverse settings to develop interventions that are effective across varied contexts. To ensure real-world applicability, further evaluation in additional patient populations is needed. We kept the maximum number of variables common to both studies, but there are certain attributes such as the number of voluntary abortions or gravidity that could have been beneficial for prediction. We decided not to perform multiple imputation, as the variables with the highest number of missing cases (online supplemental tables S1A, B) are likely crucial for accurate prediction. Imputing these values could potentially degrade the model’s credibility. However, in other contexts, where missing data cannot be avoided, imputation may be necessary. For the secondary outcome of SGA, one could argue that using the same standard for fetal weight (INTERGROWTH-21st) yields different SGA rates among both high-risk cohorts studied, and that local standards might be more suitable than a ‘one size fits all’ approach. We additionally identified SGA in IMPACT using a Spanish standard^30^ and examined the differences in predictive performance arising when comparing the two standards (see online supplemental material). Regarding outcome prediction, preterm pregnancies and perinatal death were highly imbalanced classes, with varying occurrence rates between the two cohorts. This imbalance compromises the validity and reliability of the predictive models for these specific outcomes. Moreover, the interplay between fetal growth restriction and SGA is complex and might require longitudinal and comprehensive pregnancy follow-up to unravel it.^12^ Furthermore, the potential inaccuracy of GA assessment in FeDoC could likely compromise the ability of the ML models trained on this cohort to predict SGA (see online supplemental material). Lastly, potential differences in ultrasound quality between both cohorts should not be obviated.

In future research, larger data sets would help capture more preterm and perinatal death events, potentially providing insights into their predictability, which we were not able to satisfactorily assess. Also, longitudinal models that follow the growth of the fetus over time could be key to understanding the gestation process and improving the predictions.^43^ As commonly used in clinical literature,^32^ prediction scores were reported at a 10% false positive rate. This approach aims to mitigate overflowing clinics with false positives, thereby ensuring that resources are allocated more efficiently for closer follow-up of those at real risk. In future work, a sequential approach to data analysis could provide a way forward to increase sensitivity in a more affordable and realistic setting.^44^ In this study, we showcase the effectiveness of combining fetal ultrasound and ML in the assessment of antenatal risk. Yet, for practical use in a clinical setting, ML models need to be trained with large volumes of diverse, high-quality and well-labelled data pertinent to the specific application context. Consequently, it becomes paramount to impart good fetal biometry and Doppler acquisition skills to field staff, while consistently monitoring data quality through quality check tools. This effort is pivotal in enabling ML tools to positively impact clinical decision-making during pregnancy.

## Conclusions

In summary, this paper assesses the predictability of LBW in high and low-resource scenarios, analyses the performance of external validation of the models across them and interprets model decisions considering specific input variables. The ML models created and discussed in this study allow for new insights into the pathophysiological role of the different sets of ultrasound and maternal clinical characteristics interrogated and highlight the necessity for cautious application of predictive models across geographically and income-diverse contexts. The FeDoC study illustrates the feasibility of harmonising ultrasound data acquisition and quality with standards in HIC, and our findings derived from it underscore the added value of ultrasound data to predict LBW within an LMIC beyond the maternal clinical characteristics typically used in these resource-constrained settings. We recognise the complexity of perinatal mortality as a systemic issue compounded by multiple factors that resist straightforward solutions. However, given the pressing need to reduce disparities to improve global maternal and child health—especially in regions with restricted healthcare resources such as Pakistan—our findings can be seen as progress. They demonstrate the ability of ML to tackle complex information from diverse cohorts, which could be translated into decision-support tools that would equip caregivers to identify and intervene in high-risk pregnancies. This could ultimately improve maternal and neonatal health indicators, thus reducing healthcare inequities in low-resource environments.

## Supplementary material

10.1136/bmjgh-2024-016088 online supplemental file 1

## Data Availability

Data are available upon reasonable request.
