# Supplementary material for: Prediction of low birth weight from fetal ultrasound and clinical characteristics: a comparative study between a low- and middle-income and a high-income country
Source: BMJ Glob Health. 2024 Dec 5;9(12):e016088. doi: 10.1136/bmjgh-2024-016088 (PMC11624760; doi:10.1136/bmjgh-2024-016088)
Supplement: online supplemental file 1 [file bmjgh-9-12-s001.pdf]

Supplementary Material

Discussion of local scales and Intergrowth-21

As previously reported, Intergrowth-21st standard may fail to detect at-risk SGA infants (birthweight < 10 centile), which may be especially true for western populations comprised by ethnic groups with larger maternal size (https://doi.org/10.1016/j.ajog.2015.10.931, https://doi.org/10.1002/uog.17287). In the case of IMPACT, the proportion of SGA cases using the Intergrowth-21st scale was 10.5% vs. 15.7% when using the local scale. Indeed, the SGA cases detected by both the local standard and Intergrowth-21st are the ones that are at higher risk, whereas those only detected by the local standard are somehow those cases lying in the grey zone.

At the left figure below, we show the comparison of both standards, which reveals a non-linear relation. On the right, we depict the difference between scales assessed at different GAs for individuals with the same weight. The observed differences reinforce the need for caution when applying the Intergrowth-21st tool to populations different from those on which it was trained.

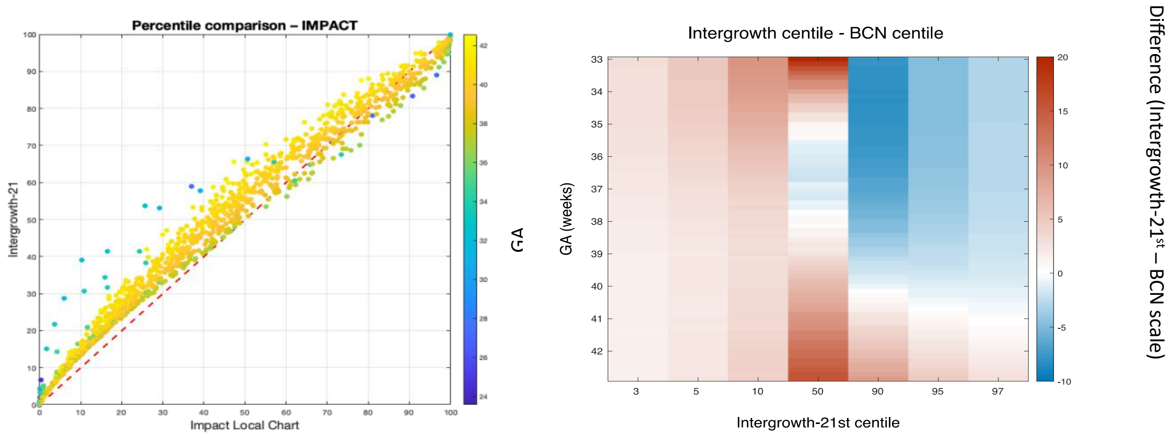

Figure S1

Despite the previously observed differences, and for the sake of comparison and generalization of a model trained on one cohort to the other, in the manuscript we report models' performance in detecting SGA encoded using the Intergrowth-21st scale in both cohorts. Below, a comparison of model's performance across all experiments. As observed, model's performance is superior when using the Intergrowth-21st scale in IMPACT, likely

because it is easier for the model to predict these higher-risk cases. In the assessment of model generalizability to the unseen cohort (IMPACT – FeDoC | FeDoC – IMPACT), the models trained at detecting SGA as encoded with the Intergrowth-21st scale in both cohorts systematically outperform those trained at detecting SGA cases encoded using different scales (Intergrowth-21st in FeDoC and Barcelona scale in IMPACT).

Flow of participants through the FeDoC and IMPACT studies

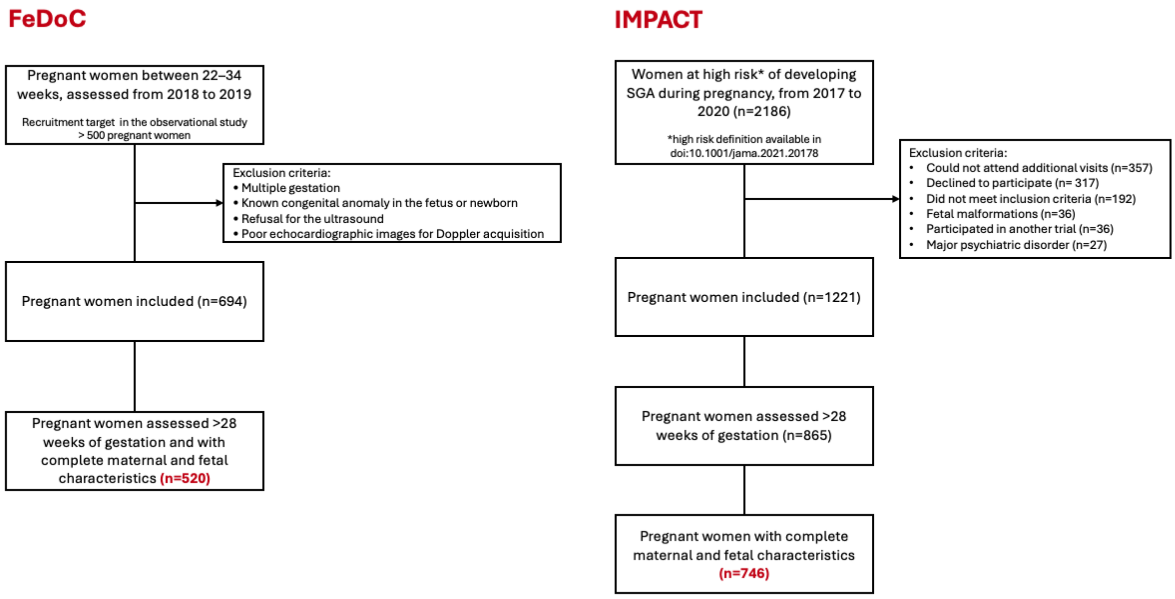

Figure S2

Proportion of missing data in both cohorts

FeDoC (n = 520)

Characteristic	Missing cases	%
Hypertensive pregnancy disorders	18	3.5%
Convulsions	3	0.6%
Gestational diabetes mellitus	4	0.8%
Anemia or iron deficiency	3	0.6%

Table S1A

IMPACT (n = 865)

Characteristic	Missing cases	%
Maternal weight	5	0.6%
Maternal SBP at visit time	33	3.8%
Maternal DBP at visit time	33	3.8%
Maternal Hb at visit time	44	5.1%
Head circumference	2	0.2%
Biparietal diameter	2	0.2%
Femur length	3	0.3%
Abdominal circumference	6	0.7%
GA at ultrasound	3	0.3%
Umbilical artery pulsatility index	7	0.8%
Middle cerebral artery pulsatility index	34	3.9%

Table S1B

Ultrasound

In brief, umbilical artery was measured from a free-floating cord loop. Middle Cerebral Artery was measured in a transversal view of the fetal head, at the level of its origin from the circle of Willis. Their corresponding pulsatility indexes were derived from focused Doppler pulsed-wave acquisitions. Cerebro-placental ratio (CPR) was calculated by dividing middle cerebral artery pulsatility index (MCA PI) by umbilical artery pulsatility index (UA PI). These spectral Doppler parameters were acquired automatically from three or more consecutive waveforms, with the angle of insonation as close to 0º as possible. Additionally, fetal biometry was obtained from routine ultrasound examination including biparietal diameter, head circumference, abdominal circumference, and femur length. These measurements were acquired thrice and averaged. In IMPACT, ultrasound was performed using Voluson E8 Expert (GE Healthcare, Illinois, USA) machine with 2-5 MHz linear curved-array probes. In FeDoC, ultrasound was performed using Vivid iq (GE Healthcare, Zipf, Austria) machine equipped with a 4.5 – 11.5 Hz phased-array transducer.

Data processing

In the FeDoC dataset, smoking, sniffing/chewing tobacco, and chewing betel nut were recoded into values of 0, 1, and 2, representing non-usage, cessation, and ongoing usage

respectively. Likewise, in the IMPACT dataset, habits such as alcohol consumption, drug use, and smoking were recoded into the same values. Each patient's risk score was then calculated as the maximum value among these risk scores. The education data from FeDoC was restructured into categories: 'no/primary' for 0-6 years of education, 'secondary/technology' for 7-12 years, and 'university' for more than 12 years, to correspond with the categories of the IMPACT survey. Similarly, FeDoC 's employment categories, which originally included 'Does not work', 'Private Job', 'Other work', 'Self-employed', 'Employed', 'Midwife', and 'Student', were simplified to align with those of IMPACT, namely 'Unemployed', 'Private/Other/Student', and 'Self-employed'.

Table S2: A comparison of model's performance at predicting SGA when using the local vs. the Intergrowth 21st scale to encode SGA in IMPACT.

		SGA (Intergr. -BCN st.)	SGA (Intergrowth)
Train IMPACT Test IMPACT	Set1	65.7 ± 4.0	59.4 ± 5.3
	Set2	74.8 ± 4.5	78.7 ± 2.0
	Set3	63.1 ± 4.2	65.8 ± 4.7
	Set4	77.6 ± 2.7	75.0 ± 3.4
	Set5	67.9 ± 5.1	67.4 ± 5.8
	Set6	75.5 ± 5.5	80.3 ± 3.0
	Set7	77.0 ± 5.4	80.3 ± 2.4
Train FeDoC Test IMPACT	Set1	54.3 ± 8.0	58.0 ± 6.2
	Set2	66.2 ± 5.4	70.1 ± 4.4
	Set3	59.8 ± 6.4	64.0 ± 4.7
	Set4	62.8 ± 4.8	68.8 ± 4.9
	Set5	59.0 ± 3.5	64.4 ± 3.3
	Set6	69.4 ± 5.4	76.8 ± 3.5
	Set7	63.8 ± 4.3	68.4 ± 6.7
Train IMPACT Test FeDoC	Set1	51.0 ± 6.7	56.3 ± 5.9
	Set2	62.9 ± 5.8	67.3 ± 5.5
	Set3	60.9 ± 4.1	61.3 ± 3.8
	Set4	57.4 ± 6.4	64.3 ± 6.1
	Set5	58.8 ± 4.3	63.6 ± 4.6
	Set6	69.3 ± 5.0	70.3 ± 4.5
	Set7	66.3 ± 4.2	71.0 ± 6.5

Table S3: Comprehensive performance metrics for low birthweight (<2.5 kg) prediction across all feature sets.

		AUC Train	AUC Test	Sn	Sp	PPV	NPV
Train FeDoC Test FeDoC	Set1	69.3 ± 2.3	65.0 ± 5.1	21.4 ± 6.6	90.4 ± 0.6	33.8 ± 6.5	82.9 ± 1.2
	Set2	65.4 ± 2.5	62.5 ± 3.9	10.4 ± 7.4	93.6 ± 4.2	nan ± nan	81.5 ± 1.2
	Set3	70.5 ± 2.0	66.8 ± 4.3	26.8 ± 7.0	90.8 ± 1.0	40.5 ± 7.6	84.0 ± 1.3
	Set4	70.8 ± 2.4	66.8 ± 4.2	20.7 ± 4.5	90.5 ± 0.6	33.8 ± 5.3	82.8 ± 0.8
	Set5	74.1 ± 2.1	70.8 ± 3.9	27.9 ± 5.5	90.5 ± 0.5	40.7 ± 3.7	84.1 ± 1.0
	Set6	70.8 ± 1.8	67.2 ± 5.8	28.2 ± 7.9	91.2 ± 1.2	42.3 ± 6.4	84.3 ± 1.4
	Set7	75.1 ± 2.2	70.2 ± 5.3	26.8 ± 8.6	90.3 ± 1.5	38.7 ± 6.9	83.9 ± 1.5
Train IMPACT Test IMPACT	Set1	74.6 ± 3.0	69.2 ± 8.8	30.7 ± 12.4	91.0 ± 2.0	17.8 ± 4.7	95.2 ± 0.7
	Set2	78.5 ± 1.2	74.5 ± 3.8	36.4 ± 9.8	90.4 ± 0.7	20.0 ± 4.3	95.5 ± 0.7
	Set3	70.9 ± 2.6	70.1 ± 6.6	28.6 ± 12.4	90.8 ± 1.1	16.5 ± 5.5	95.0 ± 0.8
	Set4	81.4 ± 2.5	76.7 ± 4.7	37.9 ± 10.1	90.3 ± 0.7	20.3 ± 4.3	95.6 ± 0.6
	Set5	75.5 ± 3.1	70.8 ± 5.7	43.6 ± 8.7	90.6 ± 0.8	23.5 ± 3.9	96.0 ± 0.6
	Set6	81.4 ± 1.6	78.1 ± 3.3	39.3 ± 13.3	90.5 ± 0.8	21.4 ± 6.4	95.7 ± 0.9
	Set7	83.0 ± 2.0	80.6 ± 4.1	47.9 ± 9.0	90.2 ± 0.8	24.5 ± 4.2	96.3 ± 0.6
Train FeDoC Test IMPACT	Set1	69.3 ± 2.3	59.2 ± 5.6	20.7 ± 9.3	90.9 ± 1.1	13.0 ± 5.5	94.5 ± 0.6
	Set2	65.4 ± 2.5	63.4 ± 10.9	22.1 ± 10.4	91.2 ± 1.2	14.3 ± 6.1	94.6 ± 0.7
	Set3	70.5 ± 2.0	73.2 ± 6.5	38.6 ± 9.1	90.4 ± 0.6	20.8 ± 3.9	95.7 ± 0.6
	Set4	70.8 ± 2.4	58.2 ± 5.0	15.0 ± 8.1	91.3 ± 1.4	10.4 ± 6.2	94.2 ± 0.5
	Set5	74.1 ± 2.1	66.8 ± 7.1	35.0 ± 13.0	91.3 ± 1.2	21.1 ± 7.3	95.5 ± 0.9
	Set6	70.8 ± 1.8	74.9 ± 5.8	41.4 ± 11.4	90.7 ± 0.9	22.6 ± 4.6	95.9 ± 0.7
	Set7	75.1 ± 2.2	71.9 ± 8.0	43.6 ± 11.3	90.4 ± 0.4	23.0 ± 4.9	96.0 ± 0.8
Train IMPACT Test FeDoC	Set1	74.6 ± 3.0	56.8 ± 6.7	13.9 ± 10.4	91.1 ± 1.4	23.4 ± 13.9	81.7 ± 1.7
	Set2	78.5 ± 1.2	62.0 ± 5.3	16.1 ± 10.3	91.3 ± 2.7	26.1 ± 12.8	82.1 ± 1.6
	Set3	70.9 ± 2.6	69.5 ± 5.2	25.7 ± 12.5	91.5 ± 2.9	nan ± nan	83.9 ± 2.0
	Set4	81.4 ± 2.5	61.2 ± 5.0	16.1 ± 9.5	91.2 ± 0.7	28.2 ± 13.5	82.1 ± 1.7
	Set5	75.5 ± 3.1	67.7 ± 7.5	17.1 ± 9.2	90.6 ± 0.8	28.4 ± 8.8	82.2 ± 1.6
	Set6	81.4 ± 1.6	71.2 ± 5.1	30.4 ± 8.2	90.3 ± 0.9	42.0 ± 7.4	84.6 ± 1.5
	Set7	83.0 ± 2.0	69.3 ± 5.5	26.1 ± 11.1	90.6 ± 0.7	37.8 ± 11.1	83.8 ± 2.0

Sn, sensitivity; Sp, specificity; PPV, positive predictive value; NPV, negative predictive value. All these metrics are calculated at 10% FPR

Table S4: AUCs for prediction of secondary outcomes in the test set

		SGA	Preterm	Cesarean section	Preterm (Exclude cesarean)	Perinatal death
Train FeDoC Test FeDoC	Set1	69.9 ± 3.9	57.4 ± 4.7	63.0 ± 5.2	59.5 ± 3.5	46.6 ± 9.8
	Set2	68.5 ± 2.8	55.2 ± 4.8	49.1 ± 2.7	50.5 ± 3.6	49.7 ± 8.3
	Set3	68.2 ± 5.0	58.1 ± 3.6	48.6 ± 4.6	55.0 ± 4.7	46.8 ± 6.8
	Set4	70.6 ± 4.9	57.7 ± 3.4	62.5 ± 5.3	59.5 ± 3.8	50.6 ± 9.9
	Set5	73.0 ± 4.0	58.2 ± 4.2	63.0 ± 4.8	63.6 ± 2.6	45.8 ± 7.0
	Set6	69.4 ± 3.4	59.0 ± 3.9	46.4 ± 4.8	55.5 ± 3.4	46.5 ± 6.9
	Set7	73.1 ± 3.6	59.8 ± 4.1	61.4 ± 3.7	61.0 ± 3.9	46.0 ± 9.6
Train IMPACT Test IMPACT	Set1	59.4 ± 5.3	75.5 ± 6.3	65.2 ± 2.9	57.5 ± 7.7	–
	Set2	78.7 ± 2.0	56.5 ± 4.5	55.2 ± 3.7	45.4 ± 17.1	–
	Set3	65.8 ± 4.7	63.5 ± 10.2	48.7 ± 2.6	55.6 ± 7.0	–
	Set4	75.0 ± 3.4	73.7 ± 8.0	65.2 ± 3.0	52.7 ± 11.5	–
	Set5	67.4 ± 5.8	79.5 ± 7.4	63.4 ± 2.4	48.9 ± 10.6	–
	Set6	80.3 ± 3.0	59.8 ± 9.6	52.1 ± 3.3	60.1 ± 10.5	–
	Set7	80.3 ± 2.4	75.1 ± 7.5	64.8 ± 2.2	50.6 ± 14.4	–
Train FeDoC Test IMPACT	Set1	58.0 ± 6.2	49.6 ± 6.9	56.8 ± 6.5	41.0 ± 16.4	–
	Set2	70.1 ± 4.4	51.1 ± 8.3	53.3 ± 2.9	44.6 ± 18.0	–
	Set3	64.0 ± 4.7	58.5 ± 13.3	50.2 ± 1.9	66.1 ± 19.9	–
	Set4	68.8 ± 4.9	53.4 ± 5.7	56.5 ± 3.9	41.7 ± 18.0	–
	Set5	64.4 ± 3.3	52.3 ± 10.1	57.4 ± 4.4	51.3 ± 24.4	–
	Set6	76.8 ± 3.5	56.7 ± 12.3	52.6 ± 4.5	67.5 ± 16.3	–
	Set7	68.4 ± 6.7	61.4 ± 11.6	56.6 ± 3.4	46.6 ± 20.6	–
Train IMPACT Test FeDoC	Set1	56.3 ± 5.9	50.4 ± 5.8	52.3 ± 5.0	48.3 ± 5.4	–
	Set2	67.3 ± 5.5	47.0 ± 3.5	50.3 ± 5.0	50.4 ± 4.1	–
	Set3	61.3 ± 3.8	54.8 ± 3.6	52.1 ± 4.3	57.2 ± 4.5	–
	Set4	64.3 ± 6.1	50.2 ± 3.9	50.1 ± 8.1	48.0 ± 5.6	–
	Set5	63.6 ± 4.6	53.2 ± 4.3	57.3 ± 5.2	55.4 ± 7.2	–
	Set6	70.3 ± 4.5	53.6 ± 4.5	54.5 ± 2.6	56.7 ± 4.5	–
	Set7	71.0 ± 6.5	53.2 ± 5.4	54.1 ± 6.0	55.4 ± 5.1	–

Discussion of prediction results for secondary outcomes

For low birthweight, IMPACT's results indicate slight prediction improvements when including clinical data and biometrics, rendering the full feature set superior. On the other hand, in FeDoC, Doppler indices are the most predictive, especially when combined with biometrics. In external validation contexts, combining Doppler and biometrics is most effective. For preterm births, IMPACT's results had moderate discriminative ability and point to clinical data as most predictive, with improvement when adding Doppler indices. Conversely, FeDoC data yielded poor predictions regardless of feature sets. External validation was poor when

predicting in FeDoC and slightly better when predicting in IMPACT. Cesarean section predictions were primarily driven by clinical data, but differences in this feature set among cohorts rendered external validation predictions poor. When focusing on preterm births not due to cesarean sections, the performance was also very poor. Finally, predictions for stillbirth and neonatal death (in FeDoC) were equivalent to random chance, indicating the limitations of the datasets used in this study.

Table S5: Comprehensive performance metrics for SGA prediction across all feature sets

		AUC Train	AUC Test	Sn	Sp	PPV	NPV
Train FeDoC Test FeDoC	Set1	72.1 ± 2.3	69.9 ± 3.9	25.4 ± 7.9	90.2 ± 0.9	35.4 ± 7.5	84.8 ± 1.4
	Set2	68.7 ± 2.0	68.5 ± 2.8	24.2 ± 4.6	90.0 ± 0.7	34.2 ± 4.0	84.6 ± 0.7
	Set3	69.2 ± 2.1	68.2 ± 5.0	20.4 ± 8.9	90.8 ± 0.7	31.2 ± 9.8	84.1 ± 1.5
	Set4	73.8 ± 2.9	70.6 ± 4.9	31.6 ± 10.0	90.3 ± 0.5	40.4 ± 8.2	85.9 ± 1.8
	Set5	75.0 ± 1.9	73.0 ± 4.0	31.6 ± 6.8	90.4 ± 0.6	41.2 ± 4.5	85.9 ± 1.2
	Set6	71.2 ± 1.7	69.4 ± 3.4	19.2 ± 6.9	90.5 ± 0.4	29.7 ± 8.3	83.8 ± 1.2
	Set7	75.6 ± 2.3	73.1 ± 3.6	28.5 ± 11.4	90.3 ± 0.6	37.5 ± 9.6	85.4 ± 2.0
Train IMPACT Test IMPACT	Set1	64.7 ± 2.6	59.4 ± 5.3	16.5 ± 8.7	90.4 ± 0.8	15.7 ± 7.7	90.5 ± 0.9
	Set2	77.3 ± 2.0	78.7 ± 2.0	37.8 ± 6.5	90.2 ± 0.3	30.5 ± 3.5	92.7 ± 0.7
	Set3	69.5 ± 2.8	65.8 ± 4.7	26.1 ± 11.8	91.5 ± 3.0	nan ± nan	91.6 ± 1.1
	Set4	77.6 ± 1.2	75.0 ± 3.4	41.3 ± 8.3	89.9 ± 0.2	31.7 ± 4.4	93.1 ± 0.9
	Set5	72.4 ± 2.9	67.4 ± 5.8	30.9 ± 8.8	90.2 ± 0.5	26.1 ± 5.8	91.9 ± 1.0
	Set6	80.5 ± 1.7	80.3 ± 3.0	44.3 ± 8.0	90.2 ± 0.7	34.0 ± 5.1	93.4 ± 0.9
	Set7	80.1 ± 1.6	80.3 ± 2.4	44.3 ± 7.0	90.1 ± 0.7	33.8 ± 3.8	93.4 ± 0.8
Train FeDoC Test IMPACT	Set1	72.1 ± 2.3	58.0 ± 6.2	16.1 ± 8.5	90.4 ± 0.7	15.5 ± 6.9	90.4 ± 0.9
	Set2	68.7 ± 2.0	70.1 ± 4.4	27.4 ± 7.3	90.4 ± 0.5	24.3 ± 5.1	91.6 ± 0.8
	Set3	69.2 ± 2.1	64.0 ± 4.7	24.3 ± 6.8	90.2 ± 0.4	21.9 ± 4.5	91.3 ± 0.7
	Set4	73.8 ± 2.9	68.8 ± 4.9	27.8 ± 8.1	90.2 ± 0.3	24.3 ± 5.0	91.6 ± 0.8
	Set5	75.0 ± 1.9	64.4 ± 3.3	20.8 ± 9.1	91.1 ± 0.8	20.4 ± 6.9	91.0 ± 0.9
	Set6	71.2 ± 1.7	76.8 ± 3.5	36.9 ± 12.0	89.9 ± 0.7	29.0 ± 6.4	92.6 ± 1.3
	Set7	75.6 ± 2.3	68.4 ± 6.7	30.9 ± 10.0	90.2 ± 0.3	26.0 ± 6.6	91.9 ± 1.1
Train IMPACT Test FeDoC	Set1	64.7 ± 2.6	56.3 ± 5.9	13.5 ± 7.2	90.4 ± 0.7	22.4 ± 9.0	82.8 ± 1.2
	Set2	77.3 ± 2.0	67.3 ± 5.5	21.2 ± 6.7	90.6 ± 0.5	32.1 ± 6.2	84.2 ± 1.1
	Set3	69.5 ± 2.8	61.3 ± 3.8	10.8 ± 7.3	93.7 ± 4.2	nan ± nan	82.9 ± 0.6
	Set4	77.6 ± 1.2	64.3 ± 6.1	19.2 ± 7.9	90.5 ± 0.8	29.4 ± 6.7	83.8 ± 1.3
	Set5	72.4 ± 2.9	63.6 ± 4.6	15.8 ± 9.0	92.0 ± 2.4	26.6 ± 12.9	83.5 ± 1.3
	Set6	80.5 ± 1.7	70.3 ± 4.5	21.5 ± 6.9	89.9 ± 0.4	30.9 ± 7.7	84.1 ± 1.2
	Set7	80.1 ± 1.6	71.0 ± 6.5	26.2 ± 10.9	90.5 ± 0.8	35.9 ± 10.3	85.0 ± 1.9

Sn, sensitivity; Sp, specificity; PPV, positive predictive value; NPV, negative predictive value. All these metrics are calculated at 10% FPR

Table S6: Comprehensive performance metrics for preterm prediction across all feature sets

		AUC Train	AUC Test	Sn	Sp	PPV	NPV
Train FeDoC Test FeDoC	Set1	60.1 ± 2.9	57.4 ± 4.7	14.0 ± 5.7	90.9 ± 1.9	35.7 ± 8.0	74.2 ± 1.2
	Set2	59.6 ± 2.5	55.2 ± 4.8	11.4 ± 6.8	92.7 ± 3.7	nan ± nan	74.0 ± 0.9
	Set3	61.8 ± 1.1	58.1 ± 3.6	19.5 ± 7.5	91.0 ± 2.9	45.9 ± 12.1	75.5 ± 1.5
	Set4	61.7 ± 3.2	57.7 ± 3.4	15.0 ± 4.1	90.5 ± 0.8	36.3 ± 6.3	74.3 ± 0.9
	Set5	63.2 ± 2.3	58.2 ± 4.2	16.9 ± 5.3	90.8 ± 0.7	39.5 ± 8.5	74.8 ± 1.2
	Set6	62.6 ± 1.9	59.0 ± 3.9	15.0 ± 6.2	91.4 ± 3.0	nan ± nan	74.5 ± 1.1
	Set7	63.6 ± 2.1	59.8 ± 4.1	14.5 ± 5.6	90.5 ± 0.7	34.9 ± 8.8	74.2 ± 1.3
Train IMPACT Test IMPACT	Set1	77.9 ± 4.6	75.5 ± 6.3	37.2 ± 15.9	91.0 ± 1.3	11.6 ± 4.6	97.8 ± 0.5
	Set2	63.8 ± 5.6	56.5 ± 4.5	4.3 ± 6.6	92.0 ± 2.0	1.5 ± 2.3	96.8 ± 0.2
	Set3	65.6 ± 4.8	63.5 ± 10.2	18.6 ± 15.7	91.2 ± 2.4	5.6 ± 4.7	97.2 ± 0.5
	Set4	75.3 ± 4.6	73.7 ± 8.0	30.0 ± 18.6	91.5 ± 2.9	nan ± nan	97.6 ± 0.6
	Set5	76.4 ± 3.7	79.5 ± 7.4	45.7 ± 21.0	90.8 ± 0.7	13.4 ± 5.2	98.1 ± 0.7
	Set6	66.4 ± 5.7	59.8 ± 9.6	14.3 ± 11.1	92.3 ± 1.7	5.3 ± 3.6	97.1 ± 0.3
	Set7	75.3 ± 3.2	75.1 ± 7.5	30.0 ± 19.6	91.0 ± 1.0	9.2 ± 5.2	97.6 ± 0.7
Train FeDoC Test IMPACT	Set1	60.1 ± 2.9	49.6 ± 6.9	4.3 ± 6.6	93.2 ± 3.1	1.6 ± 2.5	96.8 ± 0.2
	Set2	59.6 ± 2.5	51.1 ± 8.3	7.2 ± 7.2	92.7 ± 3.0	nan ± nan	96.9 ± 0.3
	Set3	61.8 ± 1.1	58.5 ± 13.3	27.2 ± 13.5	91.4 ± 1.3	9.4 ± 5.0	97.5 ± 0.4
	Set4	61.7 ± 3.2	53.4 ± 5.7	11.4 ± 12.5	92.4 ± 2.8	3.6 ± 3.8	97.0 ± 0.3
	Set5	63.2 ± 2.3	52.3 ± 10.1	20.0 ± 13.1	91.8 ± 2.3	6.7 ± 3.7	97.3 ± 0.4
	Set6	62.6 ± 1.9	56.7 ± 12.3	18.6 ± 15.7	93.4 ± 3.1	nan ± nan	97.3 ± 0.5
	Set7	63.6 ± 2.1	61.4 ± 11.6	24.3 ± 21.2	92.0 ± 2.2	7.2 ± 6.1	97.4 ± 0.7
Train IMPACT Test FeDoC	Set1	77.9 ± 4.6	50.4 ± 5.8	12.1 ± 4.8	90.6 ± 0.5	31.1 ± 8.7	73.7 ± 1.1
	Set2	63.8 ± 5.6	47.0 ± 3.5	7.4 ± 3.8	91.0 ± 1.1	22.4 ± 8.4	72.7 ± 0.8
	Set3	65.6 ± 4.8	54.8 ± 3.6	12.9 ± 4.8	91.1 ± 0.9	33.8 ± 8.4	74.0 ± 1.1
	Set4	75.3 ± 4.6	50.2 ± 3.9	9.8 ± 5.1	92.0 ± 2.9	27.8 ± 13.4	73.4 ± 0.9
	Set5	76.4 ± 3.7	53.2 ± 4.3	14.3 ± 6.5	90.7 ± 1.0	34.1 ± 9.9	74.2 ± 1.4
	Set6	66.4 ± 5.7	53.6 ± 4.5	11.9 ± 6.8	91.1 ± 2.8	28.7 ± 13.5	73.7 ± 1.3
	Set7	75.3 ± 3.2	53.2 ± 5.4	13.8 ± 4.9	90.6 ± 0.5	34.1 ± 7.9	74.1 ± 1.1

Sn, sensitivity; Sp, specificity; PPV, positive predictive value; NPV, negative predictive value. All these metrics are calculated at 10% FPR

Table S7: Comprehensive performance metrics for preterm excluding cesarean sections prediction across all feature sets

		AUC Train	AUC Test	Sn	Sp	PPV	NPV
Train FeDoC Test FeDoC	Set1	64.2 ± 3.6	59.5 ± 3.5	16.7 ± 6.8	90.6 ± 0.9	31.4 ± 7.7	80.2 ± 1.3
	Set2	56.2 ± 2.1	50.5 ± 3.6	10.0 ± 5.8	91.8 ± 2.9	nan ± nan	79.2 ± 1.0
	Set3	59.5 ± 3.4	55.0 ± 4.7	18.2 ± 5.0	90.7 ± 0.6	33.9 ± 7.4	80.5 ± 1.0
	Set4	64.7 ± 3.1	59.5 ± 3.8	17.3 ± 6.4	90.6 ± 0.4	31.9 ± 9.5	80.4 ± 1.2
	Set5	65.8 ± 2.5	63.6 ± 2.6	18.2 ± 6.8	90.6 ± 0.7	33.1 ± 8.4	80.5 ± 1.3
	Set6	58.8 ± 3.0	55.5 ± 3.4	14.2 ± 6.2	90.3 ± 0.6	27.5 ± 8.9	79.7 ± 1.3
	Set7	65.1 ± 2.1	61.0 ± 3.9	20.3 ± 9.9	90.7 ± 0.9	34.9 ± 11.5	81.0 ± 1.9
Train IMPACT Test IMPACT	Set1	74.1 ± 6.7	57.5 ± 7.7	6.7 ± 13.3	94.5 ± 3.0	0.9 ± 1.7	98.7 ± 0.2
	Set2	65.5 ± 5.3	45.4 ± 17.1	10.0 ± 15.3	91.1 ± 1.7	1.3 ± 2.1	98.7 ± 0.2
	Set3	79.0 ± 6.1	55.6 ± 7.0	10.0 ± 15.3	91.6 ± 2.3	1.4 ± 2.1	98.7 ± 0.2
	Set4	69.9 ± 9.1	52.7 ± 11.5	20.0 ± 26.7	92.8 ± 2.9	2.7 ± 3.6	98.8 ± 0.4
	Set5	78.7 ± 7.9	48.9 ± 10.6	0.0 ± 0.0	95.0 ± 3.0	nan ± nan	98.6 ± 0.1
	Set6	76.0 ± 5.8	60.1 ± 10.5	10.0 ± 15.3	92.8 ± 3.0	1.4 ± 2.2	98.7 ± 0.2
	Set7	76.4 ± 5.5	50.6 ± 14.4	13.3 ± 22.1	93.4 ± 3.1	2.3 ± 3.7	98.8 ± 0.3
Train FeDoC Test IMPACT	Set1	64.2 ± 3.6	41.0 ± 16.4	10.0 ± 15.3	95.5 ± 3.6	nan ± nan	98.7 ± 0.2
	Set2	56.2 ± 2.1	44.6 ± 18.0	6.7 ± 13.3	94.2 ± 3.0	nan ± nan	98.7 ± 0.2
	Set3	59.5 ± 3.4	66.1 ± 19.9	20.0 ± 26.7	90.8 ± 1.7	2.5 ± 3.3	98.8 ± 0.4
	Set4	64.7 ± 3.1	41.7 ± 18.0	10.0 ± 15.3	94.6 ± 2.4	1.9 ± 3.1	98.7 ± 0.2
	Set5	65.8 ± 2.5	51.3 ± 24.4	30.0 ± 34.8	93.2 ± 2.8	6.1 ± 6.9	99.0 ± 0.5
	Set6	58.8 ± 3.0	67.5 ± 16.3	23.3 ± 26.0	90.7 ± 1.0	3.4 ± 4.0	98.8 ± 0.4
	Set7	65.1 ± 2.1	46.6 ± 20.6	23.3 ± 26.0	95.9 ± 3.2	5.5 ± 6.5	98.9 ± 0.4
Train IMPACT Test FeDoC	Set1	74.1 ± 6.7	48.3 ± 5.4	10.6 ± 6.1	91.3 ± 3.0	nan ± nan	79.2 ± 0.9
	Set2	65.5 ± 5.3	50.4 ± 4.1	5.5 ± 3.0	92.7 ± 2.7	15.6 ± 9.6	78.5 ± 0.6
	Set3	79.0 ± 6.1	57.2 ± 4.5	13.3 ± 9.6	90.9 ± 1.4	24.7 ± 15.5	79.7 ± 1.7
	Set4	69.9 ± 9.1	48.0 ± 5.6	7.6 ± 4.4	90.8 ± 0.9	17.3 ± 9.2	78.6 ± 0.8
	Set5	78.7 ± 7.9	55.4 ± 7.2	14.2 ± 7.9	92.0 ± 2.8	nan ± nan	80.0 ± 1.3
	Set6	76.0 ± 5.8	56.7 ± 4.5	11.5 ± 7.5	91.6 ± 2.1	25.1 ± 10.7	79.5 ± 1.3
	Set7	76.4 ± 5.5	55.4 ± 5.1	12.7 ± 6.2	91.4 ± 2.5	28.9 ± 11.9	79.6 ± 1.1

Sn, sensitivity; Sp, specificity; PPV, positive predictive value; NPV, negative predictive value. All these metrics are calculated at 10% FPR

Table S8: Comprehensive performance metrics for cesarean section prediction across all feature sets

		AUC Train	AUC Test	Sn	Sp	PPV	NPV
Train FeDoC Test FeDoC	Set1	67.2 ± 3.1	63.0 ± 5.2	25.4 ± 10.3	90.7 ± 0.4	35.8 ± 10.9	84.8 ± 1.8
	Set2	54.7 ± 3.3	49.1 ± 2.7	8.9 ± 4.9	92.1 ± 3.3	nan ± nan	82.2 ± 0.6
	Set3	51.8 ± 2.8	48.6 ± 4.6	3.6 ± 4.5	95.1 ± 4.9	nan ± nan	81.9 ± 0.5
	Set4	66.1 ± 2.8	62.5 ± 5.3	22.5 ± 8.7	90.8 ± 0.5	33.6 ± 9.6	84.3 ± 1.5
	Set5	66.0 ± 4.0	63.0 ± 4.8	21.4 ± 9.8	91.2 ± 0.9	32.0 ± 12.8	84.2 ± 1.6
	Set6	52.3 ± 2.7	46.4 ± 4.8	2.9 ± 4.2	94.6 ± 4.4	nan ± nan	81.7 ± 0.6
	Set7	65.2 ± 3.8	61.4 ± 3.7	22.9 ± 10.4	90.8 ± 1.0	34.2 ± 11.9	84.4 ± 1.9
Train IMPACT Test IMPACT	Set1	65.3 ± 1.9	65.2 ± 2.9	25.2 ± 5.2	90.1 ± 0.5	54.7 ± 5.7	71.4 ± 1.5
	Set2	57.5 ± 3.2	55.2 ± 3.7	13.3 ± 2.9	90.2 ± 0.2	39.2 ± 4.9	68.3 ± 0.7
	Set3	51.8 ± 2.5	48.7 ± 2.6	3.6 ± 4.7	96.1 ± 4.8	nan ± nan	67.3 ± 0.4
	Set4	65.6 ± 1.9	65.2 ± 3.0	25.6 ± 7.7	90.3 ± 0.5	55.0 ± 6.2	71.6 ± 2.2
	Set5	64.3 ± 2.0	63.4 ± 2.4	20.4 ± 6.6	90.2 ± 0.4	48.8 ± 9.5	70.2 ± 1.8
	Set6	55.9 ± 2.1	52.1 ± 3.3	9.7 ± 2.9	90.6 ± 0.7	32.5 ± 5.5	67.5 ± 0.6
	Set7	65.1 ± 1.5	64.8 ± 2.2	25.2 ± 5.9	90.4 ± 0.4	55.0 ± 6.0	71.4 ± 1.7
Train FeDoC Test IMPACT	Set1	67.2 ± 3.1	56.8 ± 6.5	14.8 ± 4.1	90.3 ± 0.4	41.8 ± 7.7	68.7 ± 1.1
	Set2	54.7 ± 3.3	53.3 ± 2.9	11.4 ± 7.0	92.4 ± 3.7	nan ± nan	68.4 ± 1.2
	Set3	51.8 ± 2.8	50.2 ± 1.9	5.5 ± 6.0	95.3 ± 4.7	nan ± nan	67.6 ± 0.6
	Set4	66.1 ± 2.8	56.5 ± 3.9	13.4 ± 2.7	90.3 ± 1.3	40.0 ± 4.4	68.3 ± 0.6
	Set5	66.0 ± 4.0	57.4 ± 4.4	14.2 ± 2.5	89.9 ± 0.6	40.3 ± 4.8	68.4 ± 0.6
	Set6	52.3 ± 2.7	52.6 ± 4.5	6.7 ± 6.3	94.2 ± 4.8	nan ± nan	67.6 ± 0.8
	Set7	65.2 ± 3.8	56.6 ± 3.4	13.3 ± 3.7	90.3 ± 0.3	39.1 ± 7.4	68.3 ± 0.9
Train IMPACT Test FeDoC	Set1	65.3 ± 1.9	52.3 ± 5.0	15.0 ± 7.5	91.0 ± 1.4	25.2 ± 8.1	83.1 ± 1.1
	Set2	57.5 ± 3.2	50.3 ± 5.0	10.0 ± 3.9	91.1 ± 1.0	19.3 ± 5.9	82.2 ± 0.6
	Set3	51.8 ± 2.5	52.1 ± 4.3	5.0 ± 6.6	95.9 ± 5.0	nan ± nan	82.2 ± 0.5
	Set4	65.6 ± 1.9	50.1 ± 8.1	10.7 ± 6.8	91.2 ± 1.0	19.9 ± 10.9	82.4 ± 1.1
	Set5	64.3 ± 2.0	57.3 ± 5.2	12.9 ± 5.6	91.3 ± 1.6	24.4 ± 9.9	82.8 ± 1.0
	Set6	55.9 ± 2.1	54.5 ± 2.6	9.6 ± 6.6	91.3 ± 1.3	18.5 ± 11.5	82.2 ± 1.1
	Set7	65.1 ± 1.5	54.1 ± 6.0	14.3 ± 7.7	91.8 ± 1.4	26.2 ± 9.1	83.1 ± 1.2

Sn, sensitivity; Sp, specificity; PPV, positive predictive value; NPV, negative predictive value. All these metrics are calculated at 10% FPR

Table S9: AUCs for stillbirth and neonatal death prediction across all feature sets in FeDoC

		AUC Train	AUC Test	Sn	Sp	PPV	NPV
Train FeDoC Test FeDoC	Set1	58.9 ± 7.1	46.6 ± 9.8	9.0 ± 11.4	94.6 ± 4.9	nan ± nan	93.8 ± 0.6
	Set2	62.2 ± 8.3	49.7 ± 8.3	11.0 ± 12.2	92.5 ± 3.8	nan ± nan	93.8 ± 0.8
	Set3	56.4 ± 4.5	46.8 ± 6.8	5.0 ± 10.2	95.1 ± 4.2	nan ± nan	93.6 ± 0.6
	Set4	60.1 ± 6.0	50.6 ± 9.9	19.0 ± 9.4	91.5 ± 1.9	12.5 ± 5.9	94.3 ± 0.6
	Set5	60.5 ± 5.8	45.8 ± 7.0	7.0 ± 7.8	92.5 ± 3.2	nan ± nan	93.6 ± 0.4
	Set6	57.0 ± 5.3	46.5 ± 6.9	6.0 ± 6.6	93.8 ± 3.6	nan ± nan	93.6 ± 0.4
	Set7	57.7 ± 5.0	46.0 ± 9.6	7.0 ± 7.8	94.4 ± 4.3	nan ± nan	93.7 ± 0.5

Sn, sensitivity; Sp, specificity; PPV, positive predictive value; NPV, negative predictive value. All these metrics are calculated at 10% FPR

Discussion of baseline characteristics

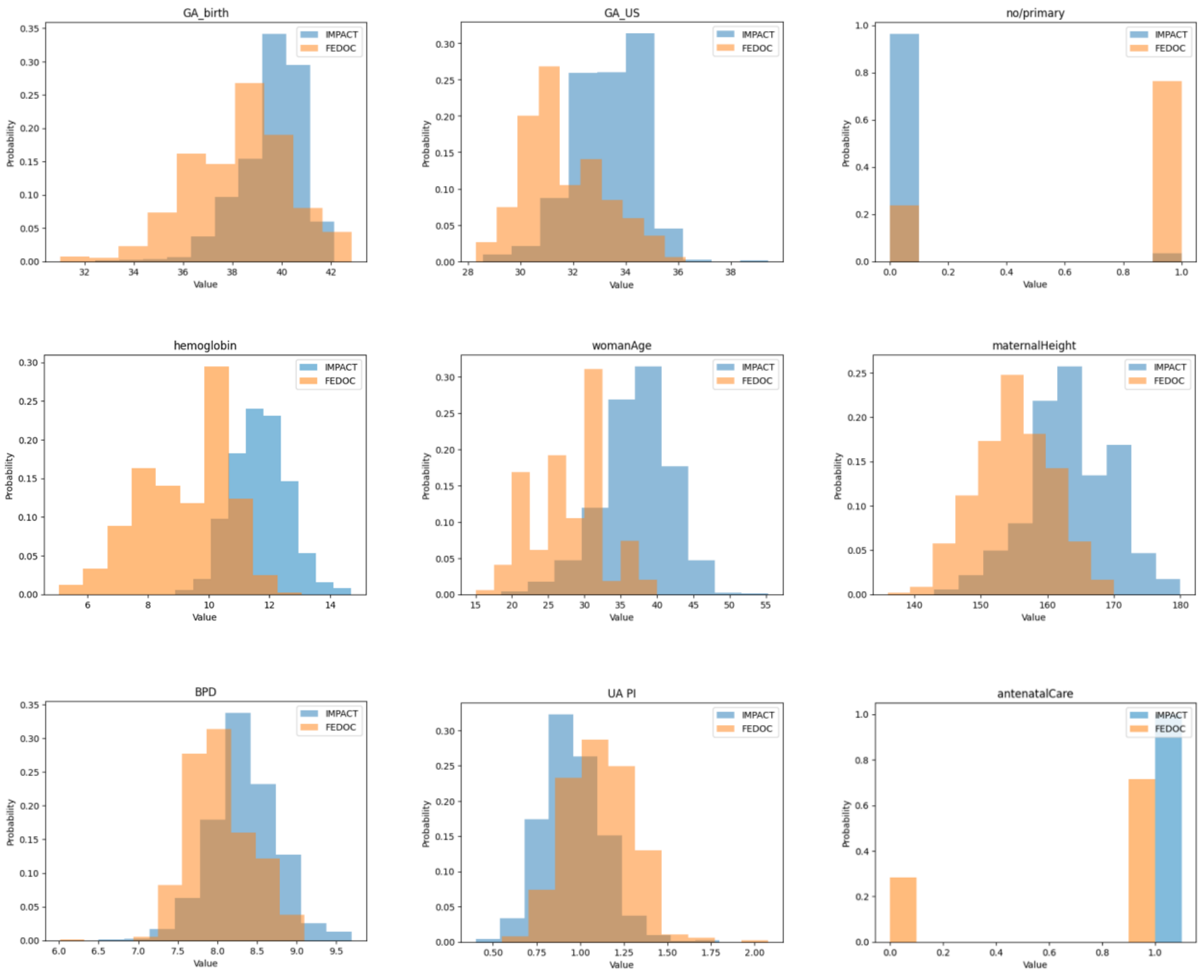

Figure S3

The cohort from Pakistan is comparatively smaller in size. This is evident across all maternal-fetal attributes, as the Pakistani women were recorded as being shorter and lighter in weight. No significant distinction was observed in blood pressure levels. The average length of pregnancies among Pakistani women was also shorter, with the mean gestational age at birth being markedly less than that of the Barcelona cohort.

Clear social disparities were detected in areas such as accessibility to antenatal care, education level, and employment status. Specifically, a third of the Pakistani women lacked access to antenatal care services, their education level was markedly lower, and a majority were unemployed. This contrasts sharply with the highly educated and near-full employment status within the Barcelona cohort.

A higher proportion of women from Barcelona halted risk-associated habits upon confirmation of their pregnancy, whereas approximately half of the Pakistani women continued these habits during pregnancy. Additionally, the Barcelona cohort exhibited a higher

representation of nulliparous women, compared to the Pakistani group, where such women were virtually absent.

Hemoglobin levels were markedly lower in the Pakistani group, falling within the range of anemia. This could be due to undernutrition and, given the amount of parous women in this group, suboptimal recovery from a previous pregnancy, among other multiple causes.

TRIPOD Checklist: Prediction Model Development and Validation

Section/Topic			Checklist Item	Page
**Title and abstract**				
Title	1	D;V	Identify the study as developing and/or validating a multivariable prediction model, the target population, and the outcome to be predicted.	1
Abstract	2	D;V	Provide a summary of objectives, study design, setting, participants, sample size, predictors, outcome, statistical analysis, results, and conclusions.	5 / 6
**Introduction**				
Background and objectives	3a	D;V	Explain the medical context (including whether diagnostic or prognostic) and rationale for developing or validating the multivariable prediction model, including references to existing models.	7
	3b	D;V	Specify the objectives, including whether the study describes the development or validation of the model or both.	8
**Methods**				
Source of data	4a	D;V	Describe the study design or source of data (e.g., randomized trial, cohort, or registry data), separately for the development and validation data sets, if applicable.	9
	4b	D;V	Specify the key study dates, including start of accrual; end of accrual; and, if applicable, end of follow-up.	9
Participants	5a	D;V	Specify key elements of the study setting (e.g., primary care, secondary care, general population) including number and location of centres.	9
	5b	D;V	Describe eligibility criteria for participants.	9
	5c	D;V	Give details of treatments received, if relevant.	9
Outcome	6a	D;V	Clearly define the outcome that is predicted by the prediction model, including how and when assessed.	10 / 11
	6b	D;V	Report any actions to blind assessment of the outcome to be predicted.	-
Predictors	7a	D;V	Clearly define all predictors used in developing or validating the multivariable prediction model, including how and when they were measured.	28 / 29
	7b	D;V	Report any actions to blind assessment of predictors for the outcome and other predictors.	-
Sample size	8	D;V	Explain how the study size was arrived at.	-
Missing data	9	D;V	Describe how missing data were handled (e.g., complete-case analysis, single imputation, multiple imputation) with details of any imputation method.	10
Statistical analysis methods	10a	D	Describe how predictors were handled in the analyses.	10 and Suppl.1
	10b	D	Specify type of model, all model-building procedures (including any predictor selection), and method for internal validation.	11 / 12
	10c	V	For validation, describe how the predictions were calculated.	12
	10d	D;V	Specify all measures used to assess model performance and, if relevant, to compare multiple models.	12
	10e	V	Describe any model updating (e.g., recalibration) arising from the validation, if done.	-
Risk groups	11	D;V	Provide details on how risk groups were created, if done.	-
Development vs. validation	12	V	For validation, identify any differences from the development data in setting, eligibility criteria, outcome, and predictors.	12
**Results**				
Participants	13a	D;V	Describe the flow of participants through the study, including the number of participants with and without the outcome and, if applicable, a summary of the follow-up time. A diagram may be helpful.	14
	13b	D;V	Describe the characteristics of the participants (basic demographics, clinical features, available predictors), including the number of participants with missing data for predictors and outcome.	14 / 28 / 29
	13c	V	For validation, show a comparison with the development data of the distribution of important variables (demographics, predictors and outcome).	Suppl.12
Model development	14a	D	Specify the number of participants and outcome events in each analysis.	28 / 29
	14b	D	If done, report the unadjusted association between each candidate predictor and outcome.	-
Model specification	15a	D	Present the full prediction model to allow predictions for individuals (i.e., all regression coefficients, and model intercept or baseline survival at a given time point).	code in GitHub
	15b	D	Explain how to the use the prediction model.	readme in GitHub
Model performance	16	D;V	Report performance measures (with CIs) for the prediction model.	30

Model-updating	17	V	If done, report the results from any model updating (i.e., model specification, model performance).	-
**Discussion**				
Limitations	18	D;V	Discuss any limitations of the study (such as nonrepresentative sample, few events per predictor, missing data).	18 / 19
Interpretation	19a	V	For validation, discuss the results with reference to performance in the development data, and any other validation data.	16 / 17
	19b	D;V	Give an overall interpretation of the results, considering objectives, limitations, results from similar studies, and other relevant evidence.	16 / 17 / 18
Implications	20	D;V	Discuss the potential clinical use of the model and implications for future research.	3
**Other information**				
Supplementary information	21	D;V	Provide information about the availability of supplementary resources, such as study protocol, Web calculator, and data sets.	Suppl.
Funding	22	D;V	Give the source of funding and the role of the funders for the present study.	22

*Items relevant only to the development of a prediction model are denoted by D, items relating solely to a validation of a prediction model are denoted by V, and items relating to both are denoted D;V. We recommend using the TRIPOD Checklist in conjunction with the TRIPOD Explanation and Elaboration document.
